# Resistance to anti-smoking messages related to the higher smoking stigma of Korean female smokers

**DOI:** 10.3389/fpsyg.2024.1427201

**Published:** 2024-09-17

**Authors:** Seung-Hyuk Ha, Gi-Eun Lee, Jang-Sun Hwang, Jang-Han Lee

**Affiliations:** ^1^Department of Psychology, Chung-Ang University, Seoul, Republic of Korea; ^2^Department of Advertising and Public Relations, Chung-Ang University, Seoul, Republic of Korea

**Keywords:** smoking stigma, public stigma, gender differences, cessation intention, implicit attitude, implicit association test (IAT)

## Abstract

**Introduction:**

The degree of perceived smoking stigma can differ, based on various factors such as gender; this may influence the effect of smoking cessation interventions, including denormalization. This study investigates the gender differences in smoking stigma recognized by Korean smokers and explores the effect of these differences on the success of smoking cessation messages that aim to initiate an identity crisis among smokers. It aims to contribute to effective smoking cessation intervention strategies for female smokers.

**Methods:**

The smoker-gender Implicit Association Test (IAT) was used to measure gender-based smoking stigma; the test comprised photos of people smoking, with positive and negative descriptors. Participants were 120 smokers aged 19–35 years (60 males and 60 females). Participants’ cognitive attitudes toward smoking and cessation intentions were assessed at baseline. To investigate the effect of social stigmatization on smokers, participants were asked to watch anti-smoking campaigns that stigmatized either smoking behavior or smokers’ self-identity. Cognitive attitudes and cessations intention were used to show differences in gender and message conditions.

**Results:**

The IAT D-score showed that female smokers perceived other female smokers significantly more negatively than they did male smokers, suggesting a higher level of smoking stigma. Female smokers in the socially stigmatizing condition reduced their negativity toward smoking less than those who were not stigmatized. Moreover, cessation intentions did not improve when female smokers received identity-threatening messages, indicating that female smokers tended to resist stigmatizing messages.

**Discussions:**

These findings provide empirical evidence that the gender of Korean smokers is significantly related to differences in smoking stigma. The negative perception and resistance responses of female smokers shown in this study are consistent with the findings of previous studies on the stigma of substance use disorders and addiction. High smoking stigma can also be a risk factor in anti-smoking interventions, including health communication; therefore, these findings should be interpreted with caution.

## 1 Introduction

The social perception of smoking in Korean society has become more negative than it used to be; the degree of smoking stigma recognized by smokers has also intensified. Recently, smoking became prohibited by law in most public places, except for certain limited zones. It has also been shown that it is regarded as an undesirable behavior by many people. During social interactions, smokers may notice a decline in their social impression, making them aware of the stigma associated with being a smoker. In contrast to other stigmatized attributes, such as physical disabilities and mental disorders, nicotine dependence is criticized more heavily and induces higher stigma among smokers themselves, as smokers continue to smoke simply due to *lack of will,* even though they have enough resources to quit smoking if they so desire (Bresnahan et al., 2013; Brown-Johnson and Popova, 2016; Heley et al., 2019). Degrading discourses on smokers are common not only among the general public but also among practitioners who provide nicotine dependence cessation interventions (van Boekel et al., 2013; Chang et al., 2016). Consequently, smokers often perceive stigma about their own smoking behavior, leading to an increased risk of depression, anxiety, low self-esteem, and reluctance to approach practitioners for cessation intervention (Asharani et al., 2020; Grant et al., 2004).

The degree of perceived stigma and its effects vary among smokers. The stigma-induced identity threat model, a theoretical framework by Major and O’Brien (2005), explains that the magnitude and effect of stigma are largely determined by the interactions of personal characteristics, situational cues, and collective representations. This model also provides a theoretical background for differences in types of stigma. Personal characteristics refer to an individual’s cognitive tendency to influence the evaluation of stigmatizing cues, such as stigma sensitivity, group identification, domain identification, goals, and motives. Situational cues indicate temporary variables that may strengthen or weaken the identity threat induced by the perceived stigma of a specific individual feature. Collective representations are features of the major groups involved in the development of public stigma. Stigmatization occurs through the interaction of the aforementioned factors, as an individual perceives stigma and identity threat appraisals. If a person determines that the magnitude of a crisis is greater than their own coping resources, the crisis is recognized as a *challenge*. If their resources are not sufficient to manage the crisis efficiently, they would recognize it as an *identity threat*. Perceived challenges or threats provoke both nonvolitional and volitional responses, including physical (e.g., increased blood pressure) and mental responses (e.g., anxiety and self-blaming). The model explains the developmental course of stigmatization and suggests that its effects may be diverse, owing to various factors. Several recent studies on nicotine dependence have reported that specific groups or cultures may experience higher smoking stigma due to the concept of group representation (Fielding-Singh et al., 2020; Helweg-Larsen et al., 2019).

In some cultural spheres, such as the Republic of Korea (hereafter, Korea), several studies have reported that a smoker’s gender is a major factor related to significant differences in smoking stigma. Research utilizing cotinine-based biomarkers has reported that the self-reported smoking rate of Korean females is significantly lower than that calculated based on biomarkers (Jung-Choi et al., 2012; Park et al., 2014). These results suggest that female smokers tend to perceive their smoking status as inappropriate, and therefore, underrate their smoking behavior in self-reported questionnaires. Qualitative research on Korean female smokers has also reported that female smokers exhibit lower self-esteem than male smokers and use maladaptive discursive strategies to deal with their gendered smoking stigma (Lee and Suh, 2006; Woo, 2018). Overall, the existing research supports the notion that female smokers perceive higher smoking stigma than male smokers. This phenomenon arises from the tendency in Korea to view female smokers more critically than their male counterparts. This tendency stems from the lingering influence of traditional Confucian values and gender role norms within the social fabric. While male smoking is somewhat socially tolerated or even seen as a symbol of masculinity, female smoking is frequently perceived as a moral failing or a sign of inadequacy in fulfilling domestic roles. This social stigma imposes additional psychological burdens on female smokers, framing their smoking not as a personal choice or a means of stress relief, but as a target for societal condemnation. Consequently, female smokers in South Korea face dual social pressures due to their smoking behavior, which can have detrimental effects on their mental health and smoking habits (Gunter et al., 2020).

Although previous research has reported significant gender differences in smoking stigma among Korean smokers, as well as its negative influences on female smokers, there are limitations regarding whether the higher smoking stigma of female smokers promotes or discourages smoking cessation. Studies from other cultural contexts, which are based on a theoretical background in the stigma-induced identity threat model, have not clearly addressed the effect of higher smoking stigma on smoking cessation (Brown-Johnson et al., 2015; Cortland et al., 2019; Helweg-Larsen et al., 2020; Kim et al., 2018). This could be due to the different characteristics of group representation shared by each culture. It is difficult to determine whether the role of smoking stigma in certain cultures manifests in an identical or similar fashion among smokers from other cultural backgrounds. Therefore, research based on data obtained from a sample of Korean smokers with identical group representations is warranted to provide culturally relevant evidence and implications for practitioners who provide treatment and intervention for Korean smokers.

Group representation is a background factor for smokers, while situational cues include the type of intervention presented. As situational cues (e.g., anti-smoking messages) interact with group representations (e.g., gender), an intervention method that has addresses smoking stigma, and its effect on the improvement of nicotine dependence should be confirmed. Denormalization is a typical anti-smoking strategy that leverages smoking stigma to convey the message that smoking is not accepted as a normal behavior and that smokers face criticism from their community (Bell et al., 2010). This method stigmatizes smokers by replacing favorable cognitive attitudes toward smoking with undesirable attitudes and thereby encourages them to stop social criticism by smoking cessation (Hammond et al., 2006). Unlike other stigma-related interventions for other conditions or situations (e.g., human immunodeficiency virus or drug use), this strategy endorses shame, rather than reducing or eliminating the stigma (Antin et al., 2015).

The tobacco denormalization approach has been adopted worldwide, and its efficiency has been proven. However, recent studies have suggested that the potential danger of this approach might override its advantages in the case of highly stigmatized groups, such as gender minority groups and smokers in low-income socioeconomic groups (Antin et al., 2015; Frohlich et al., 2012). According to Major and O’Brien (2005) stigma-induced identity threat model, this effect of vulnerable groups experiencing high stigma may lead them to evaluate denormalization messages as a threat and manage it with undesirable defense strategies. For example, highly stigmatized smokers may show a delay in discounting the negative effects on their physical health or segregating their identity from their smoking status (Brown and Faulkner, 2023; Leas et al., 2015). In summary, considering the variance in smoking stigma, denormalization strategies should be used judiciously to maximize their utility and minimize side effects.

It is crucial to distinguish between stigmatizing tobacco (or smoking behaviors) and stigmatizing smokers’ identities when stigmatization is utilized in cessation interventions. Previous research has reported that presenting negative information about tobacco has the advantage of encouraging smokers to seek help (Asharani et al., 2020). However, when smoking is associated with the identity of a smoker, this effect is diminished because the situation or message explicitly threatens the smoker’s self-identity. It may provoke an identity threat for a smoker, potentially resulting in the receiver evading the negative effect with other dysfunctional strategies, such as dissociating their identity from smoking (Leas et al., 2015). Conventional denormalization strategies in Korea, such as warning images on nicotine packages, have focused on highlighting negative images and the adverse effects of tobacco use and smoking behavior, rather than blaming smokers. These types of messages are designed to evoke fear among smokers regarding their health and are less concerned with the shame or identity threat that smoking stigma induces in the social context. Recently, an alternative approach has emerged, which involves describing the identity threat to smokers in social interactions with nonsmokers and stigmatizes smokers’ self-identity, rather than smoking behavior. As this alternative strategy is designed to directly induce identity threats among smokers, the gender difference in smoking stigma might show some advantages—or disadvantages—for the more stigmatized group. Therefore, this study included two types of negative messages: (1) individual-focused and (2) socially stigmatizing and psychological responses (attitudes toward smoking and cessation intention) in the design. Thereby, the purpose of this study was to provide detailed empirical evidence on the role of smoking stigma and the effect of stigmatizing messages on Korean smokers.

In summary, this study aimed to contribute toward improving health services related to nicotine dependence by investigating the extent of gender differences in smoking stigma among Korean smokers; it focuses on the effect of stigma on smoking cessation when using two types of stigmatizing cues. The implicit association test (IAT) was adopted to measure smoking stigma, in order to minimize the unexpected effects of social desirability and precisely quantify the degree of gender differences. The Effect on Attitudes toward Smoking was designed to measure positive and negative attitudes toward smoking as changes in the representations of smoking behavior. This study also included smoking cessation intention as a dependent variable, to represent smokers’ self-identity, and thereby provide evidence to define the role of smoking stigma on both smoking-related and smoker-related factors that may contribute to the motivation to improve their stigmatized status.

## 2 Materials and methods

### 2.1 Participants

Using G Power 3.1.9.7 (the University of Düsseldorf, Düsseldorf, Germany), a power analysis was conducted with an effect size of 0.40, an alpha error probability of 0.05, a power of 0.95, and the number of groups set to 4. The analysis showed that the minimum sample size required was 76 participants (38 participants per condition). Participants were recruited through advertisements in a campus bulletin and local online community; the advertisements provided a URL for a website where participants could access an application form and screening questionnaires. Participants were presented with the Korean versions of the Fagerström Test for Nicotine Dependence (FTND) and the Barratt Impulsiveness Scale-11–Revised (BIS-11-R). Applicants who smoked more than three cigarettes daily were recruited as current smokers, while those who scored more than 74 points (higher than +2 standard deviations) on the BIS-11-R were excluded, to improve the validity of the D-score of the IAT and a series of self-reported questionnaires. Ultimately, 120 participants, consisting of 60 men and 60 women, were included in the final sample. Participants were randomly assigned to either an individual-focused or a socially stigmatizing message condition.

### 2.2 Measurement

#### 2.2.1 Self-reported questionnaires

The FTND was used to explore the severity of nicotine dependence in each participant. It consists of six items that assess various aspects of a smoker’s cigarette smoking habit, such as the time of their first cigarette of the day, their daily cigarette consumption, and their impatience when unable to smoke in a public place (Heatherton et al., 1991). The Korean version of the FTND was validated for internal consistency, with a Cronbach’s *α* of 0.69 (Ahn et al., 2002). In the present study, it was validated with an internal consistency of Cronbach’s *α* = 0.61.

The BIS-II-R measures the degree of impulsiveness of an individual, using 30 items answered on a 4-point Likert scale (1 = Not at all, 2 = Rarely, 3 = Often, and 4 = Very often; Luengo et al., 1991). Impulsive individuals are easily distracted by instant excitement during work and have trouble in delaying small rewards to achieve long-term goals. This study used the IAT as the main task, which requires delicate cognitive work to shift the association between some concepts and attributes. Prior research utilizing the IAT suggests excluding impulsive participants to enhance result. The Korean BIS-11-R was validated, with a Cronbach’s *α* of 0.78 (Lee et al., 2012). The present study used the Korean version of the BIS-11-R for initial screening and found it to have adequate internal consistency, with a Cronbach’s *α* of 0.69.

The patient health questionnaire-9 (PHQ-9) assesses depressive mood in the past 2 weeks. It comprises nine items, all rated on a 4-point Likert scale (0 = Not at all, 1 = More than 2–3 days, 2 = More than 7 days, and 3 = Almost every day; Kroenke et al., 2001). Each item reflects the main experience or symptoms of major depressive episodes, such as decreased interest in daily life, significantly reduced or increased sleep, and psychoactive retardation or agitation. Previous studies have shown that chronic smokers are likely to be more depressed than healthy controls when they are in danger of being stigmatized for their addictive characteristics (Evans-Polce et al., 2015; Martini et al., 2002). Therefore, the current study measured depression and included it in the analysis as a potential covariate, to control for its confounding effects on negative attitudes toward smoking. This study used the Korean version of the PHQ-9, which was validated with a Cronbach’s *α* of 0.75 and found to have good internal consistency, with a Cronbach’s *α* of 0.78 (Park et al., 2010).

The Beck’s anxiety inventory (BAI) consists of 21 items that measure several types of physical and emotional symptoms of anxiety using a 4-point Likert scale (0 = Not at all, 1 = Rarely, 2 = Often, and 3 = Very often; Beck et al., 1988). In the present study, the Korean version of the BAI demonstrated internal consistency, with a Cronbach’s *α* of 0.91. Because anxiety is common among Korean smokers, this construct was a potential covariate in the research design. Like depression, increased anxiety is one of the negative psychological characteristics that has a strong relationship with chronic smoking (Monroe et al., 2021). When anxious individuals notice the crisis of being stigmatized, they are more likely to be frustrated and feel that the situation is dangerous, compared to healthy controls (Major and O’Brien, 2005).

The attitudes toward smoking scale (ATS-18) is a tool designed to quantitatively measure smokers’ psychological attitudes toward smoking. It originally consists of three subscales: (1) adverse effects of smoking, (2) psychoactive benefits of smoking, and (3) pleasure derived from smoking (Etter et al., 2000). In this study, the scale was adapted to measure two categories: positivity and negativity. This adaptation follows Kang and Han (2015), who restructured the original three subscales into dimensions of positivity and negativity when standardizing the Korean version of the scale. The negativity items address the potential losses from smoking (e.g., health, social reputation), while the positivity items cover compensatory effects such as psychological stability or concentration. Therefore, the two subscales can be seen as somewhat opposing dimensions. Most chronic smokers exhibit ambivalent attitudes toward smoking. Even when they notice the harm or danger of smoking, they continue to smoke because of certain advantages. The internal consistency of the modified ATS-18 was Cronbach’s *α* = 0.78.

#### 2.2.2 Behavioral task

*Smoker-gender implicit association test (smoker-gender IAT):* The classic version of the IAT consists of two attributes and two targets and allows participants to associate an attribute with a target (Greenwald et al., 1998). It quantifies the implicit attitude toward a specific target that a participant subconsciously perceives by comparing the means of response time (RT) to associate an attribute with one target, and then another. Participants tended to show a lower RT for comfortable association and a higher RT—and a need for more cognitive resources—when associating a target with an unfamiliar or unusual attribute. The IAT presents a simple index, the D-score, by comparing the RT of opposing conditions. This enables researchers to infer whether groups’ or conditions’ implicit attitudes differ significantly. The advantage of using the IAT is that it eliminates the unexpected effect of social desirability from the observed effect—an effect that is often inevitable with self-reported questionnaires.

Considering that this study was designed to determine participants’ attitudes toward sensitive or stigmatizing themes (e.g., smoking and gender), the confounding effect of social desirability must be controlled for by using appropriate methods such as the IAT. The IAT used in this study adopted two attributes: eight positive words (e.g., happy, pleasure) and eight negative words (e.g., terrible, nasty), as suggested by Nosek et al. (2007) in their experiment. Each adjective was translated into Korean and examined by five graduate students majoring in psychology. One target used in the IAT comprised male smokers, while the other included female smokers, both represented by images of smoking individuals obtained from SmoCuDa, a validated smoking image database (Manoliu et al., 2021). Each target featured seven images of young adult smokers from different races with neutral facial expressions to control for unexpected effects of other features such as age, race, and emotions. The sequence of the IAT consisted of seven blocks, similar to other typical IATs described in Table 1. For half of the participants, researchers presented “male smokers + positive words” for the first association, while the other half began their task with “female smokers + positive words.” This counterbalanced assignment was adopted to prevent an inappropriate order effect from disrupting the validity of the effect of the independent variables.

**Table 1:** Sequence of smoker-gender (male smoker vs. female smoker) IAT.

Block	Trials	Function	Left key (E) response	Right key (I) response
1	20	Practice	Male smoker image	Female smoker images
2	20	Practice	Positive attribute	Negative attribute
3	20	Practice	Male smoker image + Positive attribute	Female smoker image + Negative attributes
4	40	Test	Male smoker image + Positive attribute	Female smoker image + Negative attribute
5	20	Practice	Female smoker image	Male smoker images
6	20	Practice	Female smoker image + Positive attribute	Male smoker image + Negative attribute
7	40	Test	Female smoker image + Positive attribute	Male smoker image + Negative attribute

As a counterbalancing design, participants were assigned to group with either odd or even number of IAT. Even numbered IAT is the alternative version that block 4, 5, and 7 replace block 1, 2, and 4, respectively. IAT, implicit association test.

The D-score of the IAT was designed to be interpreted in such a way that a positive value indicated a positive attitude toward male smokers, while a negative value indicated a positive attitude toward female smokers. However, this was not appropriate for comparing smoking stigma related to a smoker’s gender. Therefore, the researchers converted the original D-score of female smokers by multiplying −1 by its original value and used it as a dependent variable for hypothesis testing. The converted index indicated cognitive valence for smokers with a participant’s own gender, a higher value for a more favorable attitude toward smokers with their gender, and a lower value for a more undesirable attitude with the same group.

**Figure 1:**
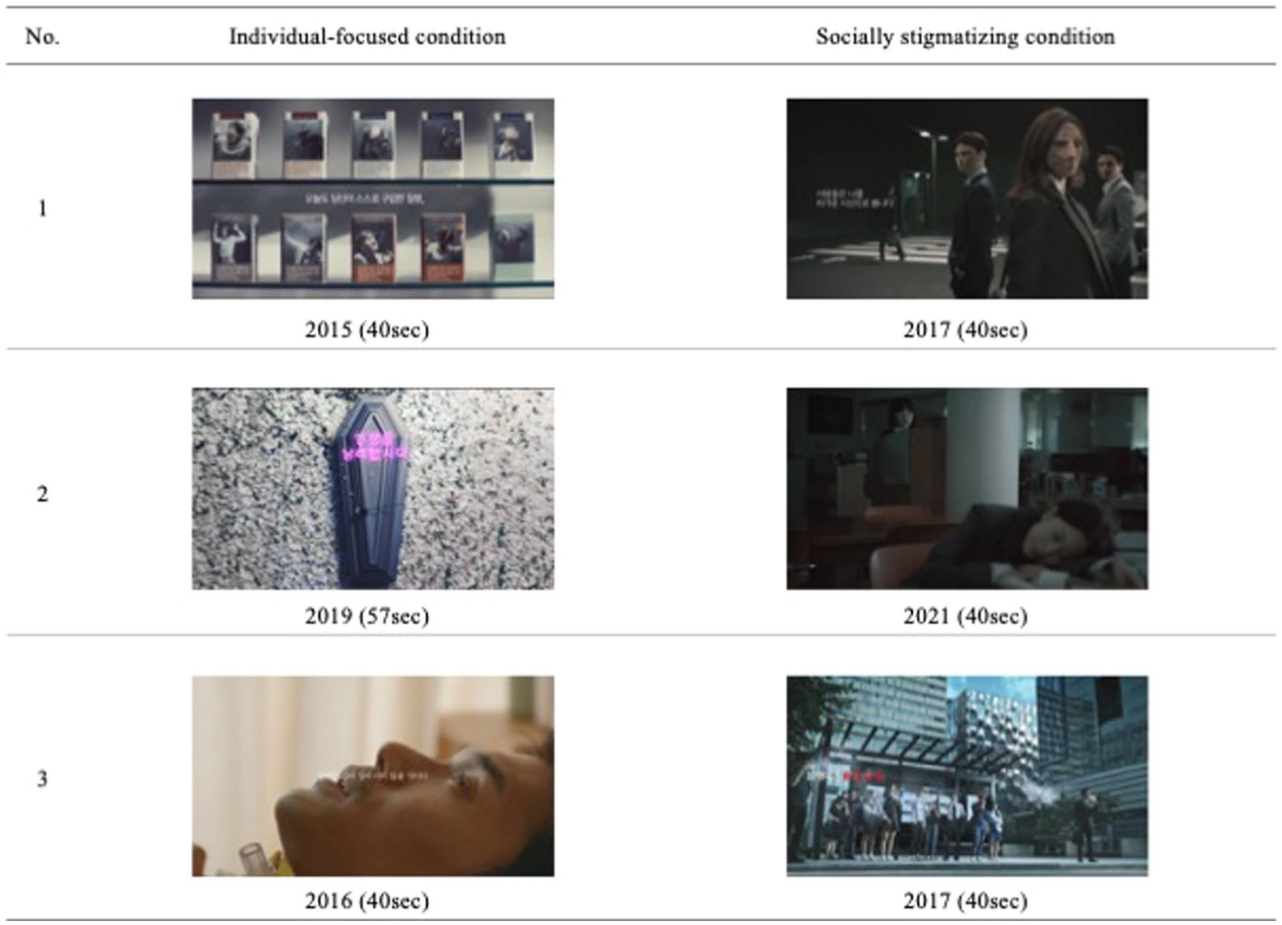
Main footages of the video clips in each condition. Source: 1 Left. “Smoking is a disease you choose to purchase”, uploaded 16 November 2015 by the Ministry of Health and Welfare of South Korea via YouTube, licensed under YouTube Standard License. 1 Right. “Anti-smoking campaign: Cold gazers”, uploaded 29 December 2017 by the Ministry of Health and Welfare of South Korea via YouTube, licensed under YouTube Standard License. 2 Left. “Shall you waste your life smoking?”, uploaded 22 July 2019 by the Ministry of Health and Welfare of South Korea via YouTube, licensed under YouTube Standard License. 2 Right. “I wish you are `no-dam’ (Ver1)”, uploaded 31 May 2021 by the Ministry of Health and Welfare of South Korea via YouTube, licensed under YouTube Standard License. 3 Left. “Anti-smoking campaign: Episode of bucket list”, uploaded 8 September 2016 by the Ministry of Health and Welfare of South Korea via YouTube, licensed under YouTube Standard License. 3 Right. “Anti-smoking campaign: Cold gazers”, uploaded 29 December 2017 by the Ministry of Health and Welfare of South Korea via YouTube, licensed under YouTube Standard License. Ministry of Health and Welfare (2015, 2016, 2017, 2019, 2021).

*Stigmatizing messages on smoking and smokers:* To trigger smoking stigma among participants, this study included the presentation of smoking cessation campaigns with negative statements. The researchers assigned participants to two types of message conditions, based on the focus of smoking-related damage: individual and social. Participants in the individual condition watched three video clips describing the physical harm caused by smoking, with the social context being completely disregarded. Contrastingly, video clips shown in the social condition described shame or identity damage caused by smoking. Each condition contained video clips of the three public cessation campaigns conducted and distributed by the Ministry of Health and Welfare (2015, 2016, 2017, 2018, 2019, 2021) (see Figure 1). Before the stigmatizing messages were delivered, the researcher noticed that the participants would write a simple review of the message. Although the review was not scored or analyzed in the present study, instructions were included to ensure that participants maintain focus on the message and to reduce cognitive avoidance of the stigmatizing message.

### 2.3 Procedures

Every participant who volunteered for the study visited a website describing the goal and warning points for the experiment and completed an application form. The first page of this form included the rights of the participants, the summarized procedure of the experiment, the guidelines for protecting personal information, and an item to confirm a participant’s willingness to engage in the research. Items for basic personal information (e.g., name, phone number, and age), FTND, and BIS-R-11 were included in the consent form for screening.

When a participant was confirmed to be suitable for the study, the researchers invited each participant to a laboratory, where they were introduced to the overall sequence that they would take part in and given the estimated time it would take to complete. Every participant initiated answering the self-reported items for potential covariates, such as the PHQ-9 and BAI. The modified ATS-18 was used as a baseline measurement of positive and negative cognitions of smoking behavior and cessation intention.

After a participant completed the pre-test measurement, a researcher introduced the IAT task and helped them prepare for the behavioral task using the practice blocks (see Table 1). Once the participants became familiar enough with the IAT, they completed the task on their own. The participants were allowed to take a short break after the IAT if they wanted to, and the researchers informed them that they must report their own feelings after watching video clips about smokers. Each participant was exposed to either individual-focused or socially stigmatizing messages, according to their assigned condition; subsequently, they reported their feelings and thoughts using three items. These discussions were not included in the main hypothesis testing but were included to ensure that the participants concentrated on the negative messages. The modified ATS-18 was used as a post-test measurement to investigate cognitive responses to stigmatizing situations. The entire procedure concluded with a debriefing, and each participant received KRW 10,000 as a reward. The study protocol was approved by the Ethics Committee of Chung-Ang University (IRB no. 1041078-202210-HR-239).

### 2.4 Data analyses

The demographic characteristics of the participants and their scores on the FTND, BIS-11-R, PHQ-9, and BAI were analyzed using ANOVA, before testing the main hypotheses. The results revealed that there was no significant difference in any of the variables. Therefore, none of these variables were included as covariates. Table 2 presents the descriptive statistics for these variables. The validity of the IAT scores was examined by using the average RT in each trial and the percentage of correct answers, fallowing the algorithm suggested by Greenwald et al. (2003). The results showed that none of the participants met the exclusion criteria; therefore, every participant’s response was included in the analysis.

**Table 2:** Demographic and group characteristic.

	Male smokers individual condition (*n* = 30)	Male smokers social condition (*n* = 30)	Female smoker individual condition (*n* = 30)	Female smokers social condition (*n* = 30)	*F*
Age	26.83(3.80)	26.73(4.02)	26.53(4.41)	27.23(3.91)	0.16
FTND	1.20(1.85)	1.57(2.03)	1.37(2.01)	1.13(1.57)	0.32
BAI	13.30(9.75)	13.07(10.40)	11.80(3.57)	18.07(9.86)	2.44
PHQ-9	3.69(2.90)	4.70(4.28)	4.90(3.57)	6.23(4.47)	2.36
BIS-R-11	60.17(8.35)	62.00(6.44)	61.50(8.00)	61.50(8.30)	0.30

Mean (standard deviation). As there was no significant group difference, any variable was not included in the statistical analysis for hypotheses testing. Range of each variable: Age: 19–35; FTND: 0–10; BAI: 0–63; PHQ-9: 0–27; BIS-R-11: 30–120.

To investigate gender differences in smoking stigma among Korean smokers, a *t*-test was conducted, with the D-score of the IAT as the dependent variable and gender (male vs. female). Cognitive responses to the stigmatizing messages were analyzed thrice, by performing a 2 (gender: male, female) × 2 (condition: individual-focused, social context) mixed ANOVA with repeated-measures dependent variables: positivity on smoking, negativity on smoking, and cessation intention.

Finally, to strengthen the study’s results, a sensitivity analysis was conducted by performing an analysis of covariance that included the variables designated as confounders in the study.

## 3 Results

### 3.1 Sample characteristics

Participants’ demographic characteristics and other psychological features are presented in Table 2. As there were no significant group differences in any of the variables, the repeated-measures ANOVA was used—rather than analysis of covariance—for hypothesis testing.

### 3.2 Gender differences in smoking stigma

The *t*-test comparing the means of the two genders’ D-scores showed that gender differences in smoking stigma were significant (*t* = −4.58, *p* < 0.01), confirming that female smokers tended to associate themselves with positive attributes at longer RT and negative attributes at shorter RT. As a higher converted D-score indicated a more positive attitude toward smokers regarding an individual’s gender, the results revealed that female smokers perceived more public stigma than male smokers, whose index was moderately positive.

### 3.3 Interaction of gender and stigmatizing condition to the cognitive attitudes toward smoking

**Figure 2:**
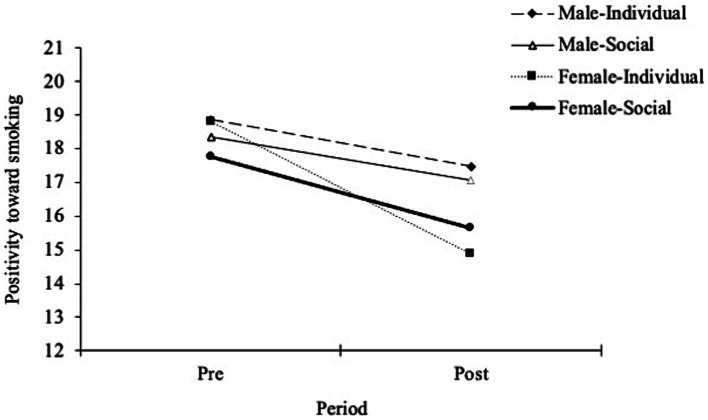
Comparison of positivity toward smoking at baseline and post-measurement in each group. Error bars represent the standard deviation of the mean.

Three repeated-measures ANOVA were conducted to investigate the interaction of gender and stigmatizing conditions, with each subscale of the modified ATS-18: positivity for smoking, negativity for smoking, and cessation intention. The first repeated-measures ANOVA conducted with positivity for smoking showed that the interaction of gender and condition was non-significant [*F*(3,116) = 0.08, *p* = 0.78, *η^2^* < 0.01], whereas the main effect of gender was significant [*F*(3,116) = 4.69, *p* = 0.03, *η^2^* = 0.03]. This result suggests that female smokers lowered their cognitive evaluation of the benefits of smoking behavior more than male smokers did in both conditions. As the within-subject analysis of baseline and post-measurement on the positivity of smoking for each subgroup revealed that the messages had a significant effect (see Table 3), both types of messages induced similar effects on depreciating positivity on smoking. The mean scores of each group, shown in repeated measurements with positivity for smoking, is presented in Figure 2.

**Table 3:** Comparison of the mean scores for positivity toward smoking in each group.

Group	Positivity toward smoking		*F*	*η^2^*
Group	Baseline	Post	*F*	*η^2^*
Male				
Individual	18.87(2.60)	17.47(3.68)	9.01^**^	0.24
Social	18.37(2.98)	17.07(3.32)	9.65^**^	0.25
Female				
Individual	18.80(2.90)	14.90(4.38)	50.37^***^	0.64
Social	17.77(2.56)	15.63(2.65)	52.47^***^	0.64

Mean (standard deviation); Test statistics (F): results of the one-way ANOVA. **p < 0.01 and ***p < 0.001.

**Figure 3:**
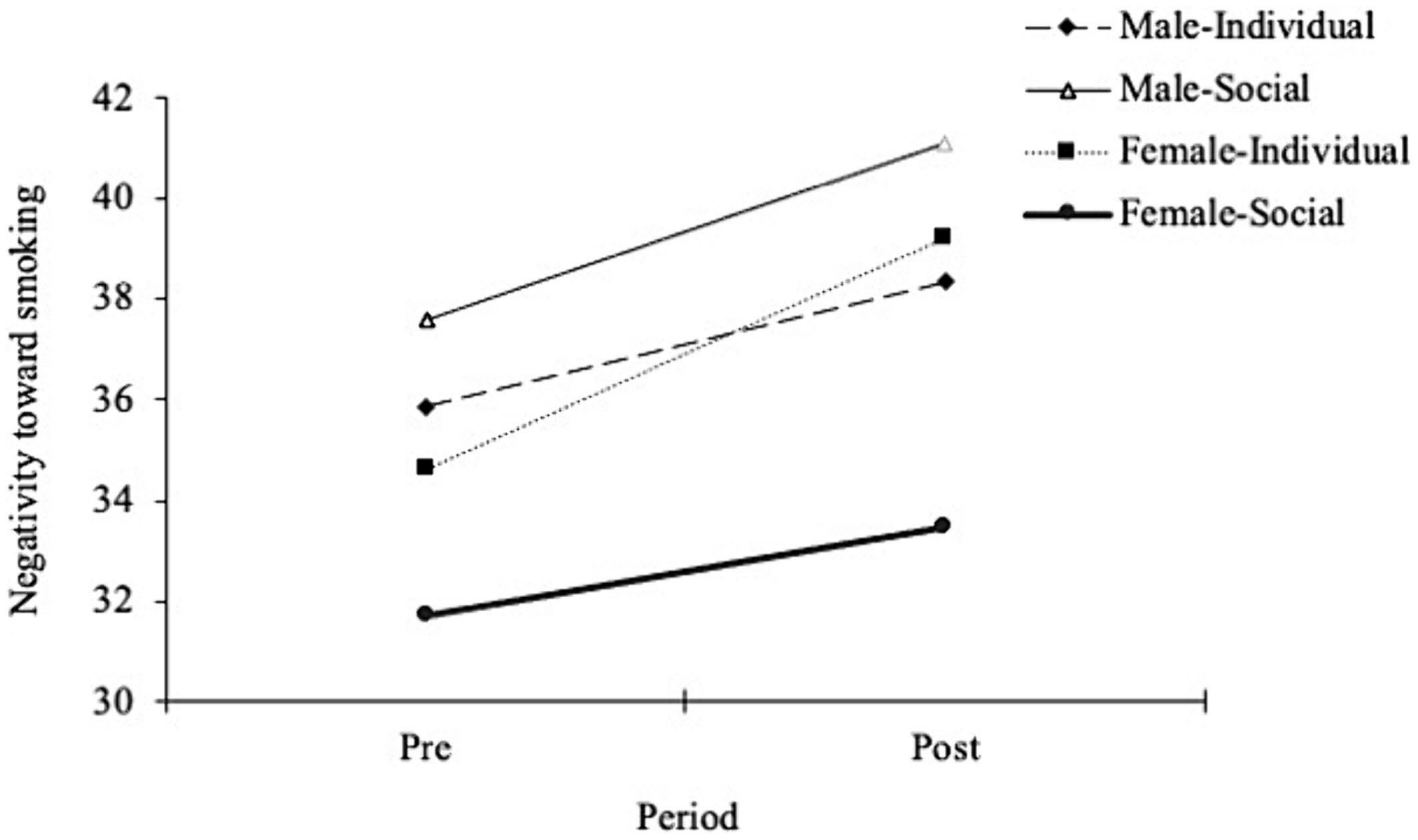
Comparison of negativity toward smoking at baseline and post-measurement in each group. Error bars represent the standard deviation of the mean.

A repeated-measures ANOVA with negativity for smoking as a dependent variable found a significant interaction between gender and condition [*F*(3,116) = 8.17, *p* < 0.01, *η^2^* = 0.05] and the significant main effect of gender [*F*(1,118) = 9.11, *p* < 0.01, *η^2^* = 0.06], whereas the main effect of condition was not significant [*F*(1,118) = 0.82, *p* = 0.37, *η^2^* < 0.01]. The simple main effect of the condition was significant in female smokers [*F*(1,58) = 6.27, *p* = 0.02, *η^2^* = 0.10], but not in male smokers [*F*(1,58) = 1.77, *p* = 0.19, *η^2^* = 0.03]. The analysis showed that female smokers tended to adjust their negative cognition of smoking behavior more in individual conditions than in socially stigmatizing conditions, whereas male smokers did not show a significant difference in either condition. Although both types of messages increased smokers’ negative cognitive attitudes toward smoking, the responses of female smokers in the socially stigmatizing condition were weaker than those in the other subgroups. Therefore, female smokers were assumed to be more resistant to anti-smoking messages, which implies the denormalization of smokers in social-interacting contexts. The comparison of negativity for smoking, to which each group responded, is described in Figure 3, and within-subject comparisons are presented in Table 4.

**Table 4:** Comparison of the mean scores for negativity toward smoking in each group.

Group	Negativity toward smoking		*F*	*η^2^*
Group	Baseline	Post	*F*	*η^2^*
Male				
Individual	35.87(6.89)	38.33(6.95)	25.27^***^	0.47
Social	37.57(5.19)	41.10(4.25)	31.08^***^	0.52
Female				
Individual	34.63(6.92)	39.20(7.44)	27.35^***^	0.49
Social	31.73(6.21)	33.50(7.71)	6.38^*^	0.18

Mean (standard deviation). Test statistics (F): results of the one-way ANOVA. *p < 0.05 and ***p < 0.001.

### 3.4 Interaction of gender and stigmatizing condition and its effect on smoking cessation intention

**Figure 4:**
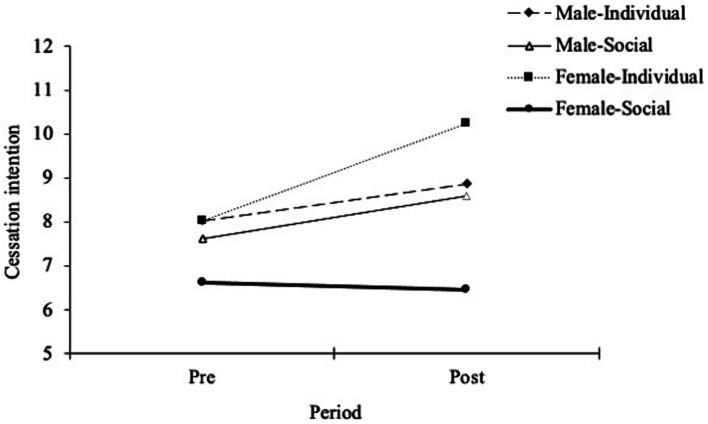
Comparison of smoking cessation intention at baseline and post-measurement in each group. Error bars represent the standard deviation of the mean.

To compare group differences in smoking cessation intentions, a repeated-measures ANOVA was performed. There was a significant interaction between gender and condition [*F*(3,116) = 4.13, *p* = 0.04, *η^2^* = 0.03] and a significant main effect on condition [*F*(1,118) = 6.94, *p* = 0.01, *η^2^* = 0.05] but a non-significant main effect on gender [*F*(1,118) = 0.63, *p* = 0.43, *η^2^* < 0.01]. The simple main effect of condition on cessation intention was significant in female smokers [*F*(1,58) = 36.65, *p* < 0.01, *η^2^* = 0.39] but not in male smokers [*F*(1,58) = 0.15, *p* = 0.70, *η^2^* = 0.03]. These results indicate that when female smokers detected smoking-stigmatizing situational cues, their motivation to quit tended to decrease—unlike male smokers under the same conditions. This suggests that female smokers adopt strategies to resist identity-threatening situations, provoking an undesirable urge to evade improvements in their stigmatized status. A comparison of the smoking cessation intentions of the different subgroups are presented in Figure 4. Table 5 presents the results of the within-subjects analysis of smoking cessation intention.

**Table 5:** Comparison of the mean scores for cessation intention in each group.

Group	Cessation intention		*F*	*η^2^*
Group	Baseline	Post	*F*	*η^2^*
Male				
Individual	8.03(3.05)	8.87(3.19)	15.04^**^	0.34
Social	7.63(3.06)	8.60(3.61)	13.34^**^	0.32
Female				
Individual	8.03(3.20)	10.23(3.04)	50.86^***^	0.64
Social	6.63(2.95)	6.47(2.75)	0.48	0.02

Mean (standard deviation). Test statistics (F): results of the one-way ANOVA. **p < 0.01 and ***p < 0.001.

### 3.5 Covariate analysis including confounders for sensitivity analysis

To strengthen the results of this study, a covariate analysis including confounders was conducted. The results indicated that excluding the confounders did not lead to significant differences. In the analysis of “positivity toward smoking,” only the effect of gender was significant [*F*(1,112) = 7.13, *p* = 0.009, *η^2^* = 0.05], and the interaction between gender and condition was not statistically significant [*F*(1,112) = 0.04, *p* = 0.84, *η^2^* = 0.00]. In the analysis of “negativity toward smoking,” both the interaction between gender and condition [*F*(1,112) = 9.26, *p* = 0.003, *η^2^* = 0.06] and the effect of gender were significant [*F*(1,112) = 8.22, *p* = 0.005, *η^2^* = 0.06], yielding results consistent with the previous analysis. For the analysis of “smoking cessation intention” both the effect of condition [*F*(1,112) = 6.07, *p* = 0.02, *η^2^* = 0.05] and the interaction between gender and condition were significant [*F*(1,112) = 5.07, *p* = 0.03, *η^2^* = 0.04], resulting in findings consistent with the earlier analysis.

## 4 Discussion

This study aimed to (1) examine the gender differences in public smoking stigma that Korean smokers implicitly perceive and (2) investigate whether stigmatizing situational cues encourage or discourage smokers who experience smoking stigma to quit smoking. First, a group comparison of the converted smoker-gender IAT D-scores revealed a significant gender difference in smoking stigma. Even if the female participants’ mean converted D-score did not show a negative value, the index was low, compared to male smokers’ index. This indicates that Korean female smokers perceived more smoking stigma. Previous research that reported gender differences in smoking stigma among Korean smokers supports using either interview methods or biomarkers (Jung-Choi et al., 2012; Lee and Suh, 2006; Park et al., 2014). It is crucial to distinguish the results of the smoker-gender IAT from previous research that adopted the smoking IAT, which used tobacco images and reported that smokers had negative implicit attitudes toward tobacco itself, rather than smokers (De Houwer et al., 2006; Swanson et al., 2001). Our findings suggest that even smokers who do not regard smoking as a pleasant behavior may show conflicting attitudes when smoking is associated with other features, such as gender. This suggests that the cognitive representations of smoking and smokers are not identical and that smokers evaluate themselves favorably, unlike smoking behavior.

This study examined the effect of two types of negative messages varying in range of focus—with and without the social-context identity threat—on the cognitive evaluation of smoking behavior. Regarding smoking positivity, negative messages significantly decreased the positive evaluation of smoking, regardless of their focus and the smokers’ gender. This means that both types of negative messages were efficient in reducing the reinforcement smokers expected from smoking. Regarding smoking negativity, both messages were found to increase the negative cognition of smoking behavior in all subgroups. However, the effect of socially stigmatizing messages on female smokers was significantly smaller than on male smokers, whereas smoking-focused messages were equally effective for all smokers. This may be because female smokers accept stigmatizing messages in a more resistant manner than male smokers under the same conditions. As female smokers in the current study perceived more stigma than male smokers, they might have tried to dissociate stigmatizing characteristics from their identity (Leas et al., 2015). Specifically, female smokers might resist stigmatizing messages that are perceived to damage their self-identity, unlike less stigmatizing male smokers, who agree with the unpleasant features of smokers. Moreover, the inefficiency of stigmatizing messages on cognitive negativity in female smokers might be because of their defensive responses to items in the modified ATS-18, which contain explicit identity-threatening features related to smoking, such as the odor of tobacco and secondhand smoke inhalation.

This study also investigated whether gender differences in smoking stigma influenced the effect of different negative messages on smokers’ cessation intentions; some caution is suggested, however, when implications are used in clinical practice and health communication. The results demonstrated that negative messages without a social context increased the intention to quit smoking in both male and female smokers. However, socially stigmatizing messages did not encourage female smokers to quit smoking; it even slightly decreased their motivation to cease smoking. These results suggest that stigmatized female smokers may opt for discursive or resistant strategies in response to the stigma-induced identity threat (Major and O’Brien, 2005; Woo, 2018). This type of response may explain the dissociation between the representation of smoking and the smokers’ self, which was identified in the gap between the results of the smoking and smoker-gender IATs (De Houwer et al., 2006). Unlike individual-focused messages that did not provoke identity threats, socially stigmatizing messages compelled smokers to consider identity threats and determine whether the situation was a challenge or a threat. Smokers who perceived it as a threat were likely to adopt unhealthy defensive strategies, disregarding negative aspects and denying them. The unique resistance to stigmatizing situational cues found in female smokers confirmed that smoking stigma related to unfavorable collective representations might impede the progress of stigmatized individuals, solidifying their acknowledgment of the imposed stigma (Evans-Polce et al., 2015; Frohlich et al., 2010).

Our findings also verified the results of self-stigma in substance use disorders (SUDs) and label avoidance—a maladaptive behavior frequently induced by addiction-related stigma (Corrigan et al., 2017). Self-stigma is a negative perception of oneself generated by internalizing devaluing discourses as a shameful feature (Link, 1987). The converted D-scores from the IAT showed that female smokers developed self-stigma by internalizing the negative social impression of the community, confirming the results of several studies on Korean female smokers (Jung-Choi et al., 2012; Lee and Suh, 2006; Park et al., 2014). Furthermore, label avoidance—the behavior of refusing treatment to avoid the negative effects caused by the label—could explain the change in the cessation intention of female smokers in socially stigmatizing conditions (Clement et al., 2015). Although the change was not statistically significant, the responses of female smokers to stigmatizing cues were similar to those of patients with alcoholism and other SUDs (Keyes et al., 2010; Rivera et al., 2014). Therefore, the higher smoking stigma and resistance response pattern of female smokers in the current study reaffirmed the stigma of SUDs and nicotine dependence.

Despite its clear contributions, this study has some limitations. Although the study adopted a baseline and post-measurement design, the IAT for smoking stigma was not included in the post-measurement stage, as the behavioral task was not suitable, owing to the carry-over effect. However, we attempted to compensate for the lack of multiple implicit measurements with the negativity of smoking, which partially addressed the negative impressions of shameful smokers. Future studies should adopt a suitable design to examine changes in smoking stigma post-manipulation. Another limitation is the exclusion of impulsive smokers, who are common in the smoker population, because of the complicated procedure. Impulsive smokers would show different interactions with the stigmatizing or negative messages presented in the experiment, which would hinder the ecological validity of the findings. We recommend that future studies simplify the screening questionnaires or impose brief and valid methods suitable for impulsive smokers. Lastly, this study included only denormalization appeals as situational cues for smoking cessation. We recommend that, in the future, alternative strategies such as humor and acceptability be explored, based on their positive appeal.

Despite these limitations, the present study offers fundamental implications for smoking cessation treatment by health service providers and clinical practitioners. This study adopted a behavioral measurement design for implicit attitudes and provides empirical evidence of gender differences in smoking stigma, thus minimizing the unexpected effects of social desirability biases. Moreover, the undesirable effect of stigmatizing vulnerable groups was reported in this research, suggesting that health service providers should be cautious when managing group-specific stigma. Previous research on the treatment of lung cancer has suggested that thoughtless utterances by providers may frustrate patients and make it difficult for them to succeed via cessation interventions (Carter-Harris, 2015; Williamson et al., 2020). Moreover, this study’s results of individual-focused messages suggested that modifying a maladaptive belief or representation of smoking would be more helpful than taking the risk of aiming for a sensitive representation of smokers themselves, in practice. Another type of major cessation intervention is related to health communication, and the findings of this research could be applied to improve denormalization. Although some previous studies have reported that conventional fear appeals (e.g., warning graphics on cigarette packs) have a limited effect on cessation in Korea (Lee et al., 2018), they still serve as powerful motivators for smokers to quit. The effect of individual-focused messages on attitudes toward smoking and cessation intention in the present study suggests that fear appeals are not outdated or inappropriate strategies. This study also showed that using stigmatizing messages describing shameful social interactions as an alternative strategy may have potential risks that do not exist in traditional messages. Finally, our results show that gender differences in smoking stigma may be a latent risk factor in any culture or group and should be considered in interventions aimed at reducing nicotine dependence.

## Data Availability

The raw data supporting the conclusions of this article will be made available by the authors, without undue reservation.
